# The importance of assessing and addressing mental health barriers to PrEP use during pregnancy and postpartum in sub‐Saharan Africa: state of the science and research priorities

**DOI:** 10.1002/jia2.26026

**Published:** 2022-10-17

**Authors:** Amelia M. Stanton, Conall O'Cleirigh, Lucia Knight, Dvora L. Joseph Davey, Landon Myer, John A. Joska, Kenneth H. Mayer, Linda‐Gail Bekker, Christina Psaros

**Affiliations:** ^1^ Department of Psychological and Brain Sciences Boston University Boston Massachusetts USA; ^2^ Massachusetts General Hospital Boston Massachusetts USA; ^3^ Fenway Health Boston Massachusetts USA; ^4^ Harvard Medical School Boston Massachusetts USA; ^5^ School of Public Health University of Cape Town Cape Town South Africa; ^6^ Division of Infectious Diseases, Geffen School of Medicine University of California Los Angeles Los Angeles California USA; ^7^ HIV Mental Health Research Unit, Division of Neuropsychiatry, Department of Psychiatry and Mental Health University of Cape Town Cape Town South Africa; ^8^ Beth Israel Deaconess Medical Center Boston Massachusetts USA; ^9^ Desmond Tutu HIV Center University of Cape Town Cape Town South Africa

**Keywords:** barriers, mental health, postpartum, pregnancy, PrEP, sub‐Saharan

## Abstract

**Introduction:**

Pregnant and postpartum women (PPW) in sub‐Saharan Africa are at disproportionately high risk of HIV infection compared to non‐pregnant women. When used consistently, pre‐exposure prophylaxis (PrEP) can prevent HIV acquisition and transmission to the foetus or infant during these critical periods. Recent studies have demonstrated associations between mental health challenges (e.g. depression and traumatic stress associated with intimate partner violence) and decreased PrEP adherence and persistence, particularly among adolescents, younger women and women in the postpartum period. However, mental health is not currently a major focus of PrEP implementation research and programme planning for PPW.

**Discussion:**

PrEP implementation programmes for PPW need to assess and address mental health barriers to consistent PrEP use to ensure effectiveness and sustainability in routine care. We highlight three key research priorities that will support PrEP adherence and persistence: (1) include mental health screening tools in PrEP implementation research with PPW, both to assess the feasibility of integrating these tools into routine antenatal and postpartum care and to ensure that limited resources are directed towards women whose symptoms may interfere most with PrEP use; (2) identify cross‐cutting, transdiagnostic psychological mechanisms that affect consistent PrEP use during these periods and can realistically be targeted with intervention in resource‐limited settings; and (3) develop/adapt and test interventions that target those underlying mechanisms, leveraging strategies from existing interventions that have successfully mitigated mental health barriers to antiretroviral therapy use among people with HIV.

**Conclusions:**

For PPW, implementation of PrEP should be guided by a robust understanding of the unique psychological difficulties that may act as barriers to uptake, adherence and persistence (i.e. sustained adherence over time). We strongly encourage PrEP implementation research in PPW to incorporate validated mental health screening tools and ultimately treatment in routine antenatal and postnatal care, and we stress the potential public health benefits of identifying women who face mental health barriers to PrEP use.

## 1 INTRODUCTION

Women remain at high risk of HIV acquisition and are the most affected by the global HIV epidemic. In sub‐Saharan Africa (SSA), 59% of new adult infections occur among cisgender women [1]. This risk is compounded during pregnancy and postpartum, when the probability of HIV acquisition increases. In serodiscordant couples, women in late pregnancy and in the early postpartum period are three to four times more likely to acquire HIV per condomless sex act than women who are not pregnant [2].

Interaction of biological and behavioural factors exacerbates risk during these periods [2, 3, 4, 5, 6, 7]. Couples may be more likely to engage in condomless vaginal and anal sex during pregnancy, as contraception may drive condom use [8, 9, 10, 11], and men may seek out other sexual partners during extended periods of pregnancy‐ or breastfeeding‐related abstinence, increasing the risk for HIV transmission when sexual activity with the pregnancy partner resumes [10]. Moreover, biological changes, such as high levels of oestrogen and progesterone, induce changes in the genital tract, increasing susceptibility to HIV and sexually transmitted infections (STI) infection [4, 12].

In areas of high HIV prevalence, cisgender women across the lifespan are at elevated HIV risk, unless they are in a mutually monogamous relationship with a known HIV‐uninfected partner. Women who are not in relationships that fit this description are in an important target group for pre‐exposure prophylaxis (PrEP), especially if they have the potential for pregnancy. In SSA, women do not always have full agency to use condoms and/or engage in other HIV prevention behaviours [13]; PrEP offers the ability to prevent HIV if sexual activity with a non‐monogamous or serostatus unknown partner cannot be avoided.

Once daily oral tenofovir disoproxil fumarate co‐formulated with emtricitabine as PrEP is safe when taken during pregnancy [14, 15, 16, 17, 18, 19, 20], recommended by the World Health Organization [21], and has extensive public health benefits. Prioritizing oral PrEP for women in the general population, as opposed to focusing PrEP rollout on women at highest risk (e.g. sex workers), could avert substantially more infections [22]. Models predict that PrEP use in pregnant and postpartum women (PPW) will reduce new HIV infections in South Africa alone by up to 7.2% by 2030 [23]. With global breastfeeding targets of 24 months and beyond [24, 25, 26, 27, 28], postnatal transmission is a growing percentage of all HIV transmission [2, 7, 29], accounting for 75% of all perinatal transmission in 2018, up from 40% in 2005 [30, 31]. Accordingly, strengthening the uptake of and adherence to effective prevention strategies like PrEP during the postpartum transition is of high importance.

## 2 DISCUSSION

### 2.1 PrEP uptake, adherence and persistence challenges during pregnancy and the postpartum period

When PrEP is offered to PPW in research studies, rates of uptake range but are relatively high. In two ongoing South Africa‐based cohorts, one that enrolled participants in antenatal care (ANC) [32] and one that recruited women planning for pregnancy [20, 33], PrEP initiation rates were approximately 90% and 60%, respectively (though initiation rates appear to be lower in Kenya at 21.7% [34]). With the high utilization of ANC across SSA (78.9%) [35], these services are an opportune time to introduce PrEP and expand implementation.

PrEP adherence rates are low in African women of reproductive age. In a recent study, only one in five participants had high adherence (defined as tenofovir diphosphate concentrations [TFV‐DP] ≥ 700 fmol/punch) over the first 6 months [36]. Importantly, differences in TFV‐DP concentrations in vaginal versus rectal tissue [37, 38] suggest that women need to be *more* adherent to maintain the same level of protection as men who have sex with men [39]. This is especially true during late pregnancy when TFV‐DP concentrations are one‐third lower than in the postpartum period [40, 41], indicating that daily PrEP use is necessary for prevention‐effective adherence during pregnancy [42, 43].

PrEP persistence, or sustained PrEP adherence over time, is crucial during pregnancy and the postpartum transition. Recent studies among PPW have reported persistence rates of 75% and 62% at 1 and 3 months post‐initiation [32]. Rates in adolescent girls and younger women who become pregnant are likely lower; only a third of adolescents and younger women in Kenya who initiated PrEP continued use after 1 month [34, 44]. The likelihood of PrEP persistence is also demonstrably lower among postpartum women at 3‐month post‐initiation (aOR = 0.31; 95% CI = 0.16–0.61) [45]. Persistence can be especially difficult post‐delivery, when the routine is disrupted and concealing PrEP use from partners is complicated by fears of perceived infidelity or promiscuousness (unlike during pregnancy, when pill‐taking is expected) [46]. Noted barriers to PrEP adherence among pregnant and non‐pregnant African women likely also have negative impacts on persistence through the postpartum transition. These barriers include structural factors, low familial or social support, anticipated HIV‐related stigma, side effects, fears of negative health consequences for the infant and mental health challenges [46, 47, 48, 49, 50, 51, 52, 53, 54, 55, 56, 57, 58]. Relative to other barriers, mental health has received little attention and has high relevance for PPW.

Notably, new biomedical HIV prevention products, including long‐acting injectables and the dapivirine ring, may benefit PPW with mental health barriers to oral PrEP use. Both injectable cabotegravir and the dapivirine ring appear to be safe for use during pregnancy [59, 60]. In addition to ensuring adequate representation of cisgender women and PPW in research on PrEP modalities that are in the pipeline and/or already in clinical trials [61], we must assess the degree to which the absence of daily dosing may mitigate or potentiate mental health‐related adherence challenges. Though the focus of the discussion below is on oral PrEP, the mental health considerations that we highlight are likely relevant to other PrEP formulations.

### 2.2 Mental health, pregnancy and consistent PrEP use

Many PPW in SSA face unique psychological and psychosocial challenges to PrEP adherence and persistence. Intimate partner violence (IPV), associated post‐traumatic stress and depression are related to reduced PrEP adherence during this high‐risk period [47, 48, 49, 50, 51]. Reported by up to 57% of pregnant women in SSA [62], IPV is a strong predictor of both post‐traumatic stress and depression [63, 64]. Women who report IPV or depression have an increased risk of low PrEP adherence [47, 48, 49, 50, 51], and IPV‐related stress contributes to adherence challenges [47]. In an analysis of depression symptom trajectories among young women initiating PrEP, 48.5% had persistent elevated depression symptoms; these women were more likely to report IPV and less likely to have detectable TFV‐DP throughout follow‐up than participants with mild or no depression symptoms [65]. Although only IPV, post‐traumatic stress and depression have been linked to low PrEP adherence and persistence in the published literature, PPW with other mental health disorders and concerns—anxiety, suicidality and alcohol use disorder are common [66, 67, 68]—likely also face challenges to consistent PrEP use. PPW with approach‐oriented coping skills, social support and high levels of resilience may be able to effectively navigate the complex barriers to PrEP adherence and persistence, but these relationships have not yet been assessed.

Mental health barriers to PrEP adherence and persistence may be particularly important to address among adolescents and young adults (AYAs), who are more likely to have unplanned pregnancies than older women [69]. Unplanned pregnancies are common in SSA (29%) and can have severe consequences [70, 71], including depression and suicide, stigma, sexual/romantic relationship challenges, violence and abuse, and lack of emotional support [72]. Any one of these factors may significantly compromise consistent PrEP use. In AYAs, the relationship among poor mental health, early and unplanned pregnancy, and risk for HIV acquisition has been conceptualized as a “syndemic,” or clustering of risk factors that together increase vulnerability to negative health outcomes above and beyond the effect of any one risk factor alone [73, 74]. With these intersecting challenges, it may be particularly difficult for AYAs with unplanned pregnancies to maintain PrEP adherence and persistence.

The integration of mental health screening and treatment into ANC has been a priority in SSA for at least a decade, with the understanding that addressing mental health in this context would ensure that gains in maternal and child health as well as the expansion of HIV services are maintained [75]. A multilevel approach to managing mental health concerns in antenatal and postpartum care is likely required [76]; this might include health promotion and primary/secondary prevention efforts in community and healthcare settings, identification and management of mental health concerns integrated into routine maternal and child healthcare by supervised non‐specialist workers, and access to specialist mental health services if needed. As implementation research identifies the most efficient and effective strategies to achieve these goals, the importance of aligning mental health integration with HIV prevention cannot be understated.

### 2.3 Research priorities and opportunities

**Figure 1:**
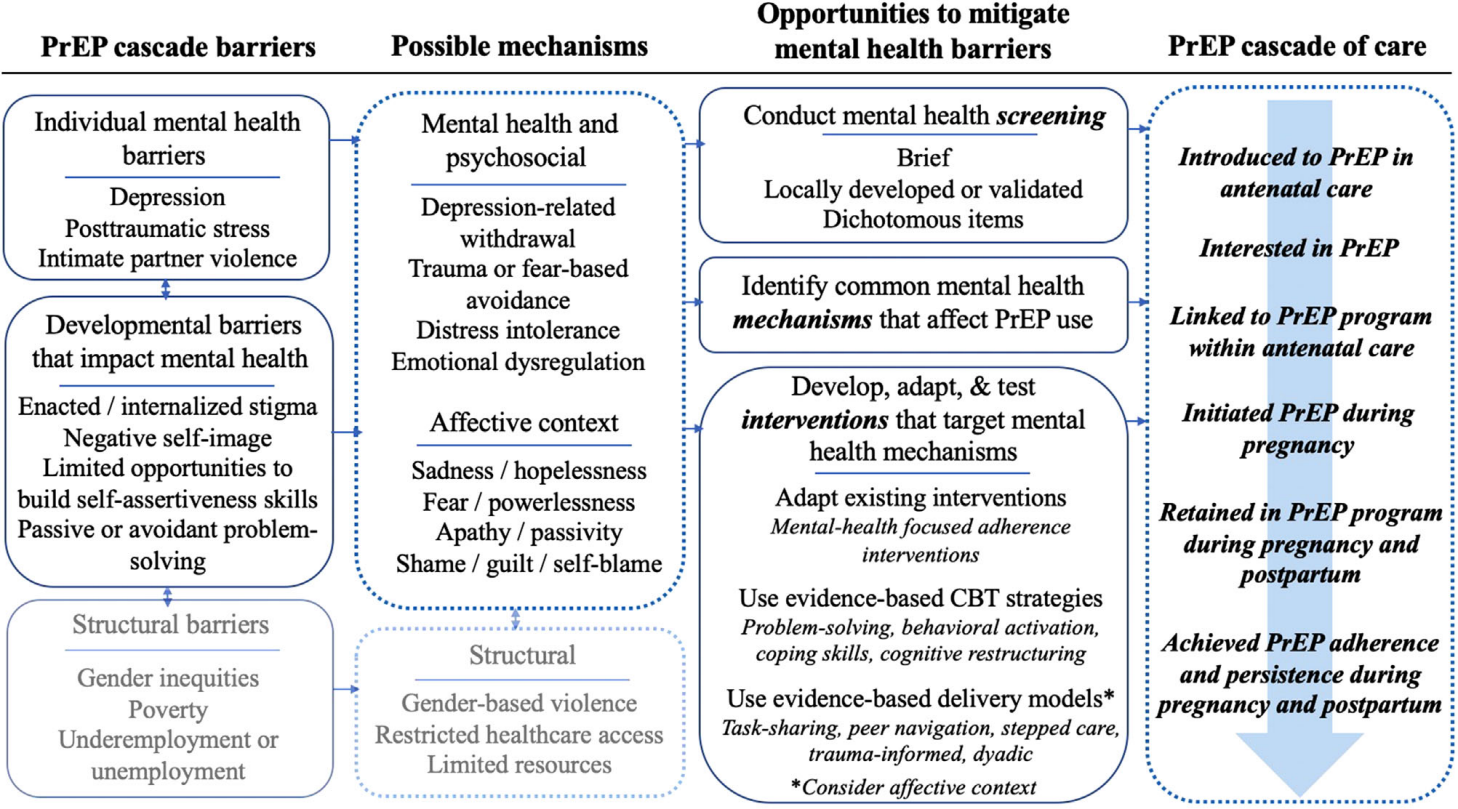
PrEP cascade of care in antenatal/postpartum care and opportunities to address mental health barriers to PrEP use.

To facilitate PrEP implementation for perinatal populations [77], we highlight three key research priorities for assessing and addressing mental health barriers to PrEP. Figure 1 presents these priorities as opportunities, documents individual mental health and associated developmental barriers (as well as structural barriers, which we do not address), offers relevant psychosocial mechanisms (with consideration for the affective context) and maps these categories onto the PrEP cascade for PPW.

#### 2.3.1 Include mental health screening tools in PrEP implementation research with PPW

Although screening guidelines for depression, for example, have been established for PPW in high‐income countries [78, 79], there are limited evidence‐based screening models for depression and other mental health disorders among PPW in SSA. Providers cite concerns about the acceptability and benefits of routine screening, the limitations of screening tools, the lack of access to follow‐up services and the added cost of both screening and treatment [80, 81, 82]. Given these concerns, it is important to extend the assessment of perinatal mental health screening tools beyond diagnostic validity to include characteristics that streamline implementation and ensure sustainability (e.g. cultural appropriateness, acceptability, question structure and length). These characteristics can be measured and compared across tools to facilitate informed screening choices that make the best use of limited resources.

Tools like the Edinburgh Postnatal Depression Scale [83] and the Patient Health Questionnaire‐9 [84] have technically been validated among PPW across SSA [83, 85, 86], but they may not be feasible for routine care. Their length (9–10 items) and reliance on Likert‐style scoring may cause confusion, negatively affect the accuracy of responses and compromise implementation by non‐specialist health workers [87, 88]. These concerns have led to an increased focus on (1) ease of implementation [89], prioritizing brevity (e.g. four items) and binary response options [90, 91], and (2) high specificity [92] to minimize costs spent on “false positives” (i.e. PPW for whom mental health is not negatively impacting PrEP use). Locally developed and validated screening tools that meet these criteria will both identify women with psychological vulnerabilities for low adherence and persistence, allowing for limited resources to be directed to those who need them most, and facilitate the integration of screening into perinatal care. There are already several tools that fit this description but may need to be adjusted for ease of use. Both the 14‐item Shona Symptom Questionnaire [93], developed to screen for depression and anxiety in Zimbabwe [94, 95], and the 16‐item South African Depression Scale [96] could be shortened and tested among PPW, as can tools that have been developed for patients with low literacy levels [97]. As SSA continues to cope with the spread of COVID‐19, with corresponding challenges to community mental health, advancing our understanding of mental health barriers to PrEP use via appropriate screening among PPW is particularly timely.

#### 2.3.2 Identify cross‐cutting, transdiagnostic psychological mechanisms that affect PrEP use and can realistically be targeted with intervention in resource‐limited settings

There has been little to no attention paid to the specific ways in which underlying psychological mechanisms may impact PrEP use among PPW. Behavioural scientists should work to identify the mechanisms that are linked to low PrEP adherence. For example, from a behavioural perspective, both post‐traumatic stress and depression are maintained by withdrawal and avoidance [98]. Individuals with post‐traumatic stress avoid experiences that elicit painful memories [98], and individuals with depression withdraw from life activities due to unremitting sadness and loss of pleasure [99]. In both cases, avoidance and withdrawal interfere with proactive self‐care behaviours [100], including taking PrEP.

Other mechanisms that may be driving the associations among depression, post‐traumatic stress and low PrEP adherence include distress tolerance [101] and emotion regulation. PPW with low distress tolerance may not be able to manage the negative affect states (e.g. sadness and fear) that accompany depression or post‐traumatic stress, and/or they may not be able to navigate the distressing, intersecting barriers associated with refilling their prescriptions and continuing to take PrEP time. Similarly, women who lack emotion regulation skills and are experiencing negative self‐conscious emotions (e.g. shame and guilt) that are associated with post‐traumatic stress and depression may also struggle to take PrEP.

#### 2.3.3. Develop/adapt and test interventions that target those underlying mechanisms

Targeting multiple, cross‐cutting psychological mechanisms—including withdrawal, avoidance, distress tolerance and emotion regulation [102, 103, 104, 105, 106, 107]—in PrEP use interventions for PPW will streamline implementation. Transdiagnostic mental health interventions (e.g. the Unified Protocol [108], the Common Elements Treatment Approach [109]) capitalize on the similarities of symptoms across disorders and components of evidence‐based programmes; reduce the number of models in which non‐specialist providers need to be trained; and reduce the cost of that training [110, 111, 112, 113]. These benefits are highly relevant to SSA.

Existing interventions and evidence‐based intervention strategies that target post‐traumatic stress and depression as pathways to improved antiretroviral therapy (ART) adherence among people with HIV can be adapted for PrEP use among PPW. For example, elements of a trauma‐focused ART adherence intervention developed for South African women with histories of sexual violence can be leveraged; promising pilot data showed decreases in post‐traumatic stress and increases in ART adherence motivation in the intervention [114], and a larger randomized controlled trial (RCT) is currently underway (NCT04793217). Similarly, a pilot study of cognitive behaviour therapy (CBT)‐based intervention for ART adherence and depression led to decreased depression among postpartum women with HIV [115], and a separate RCT demonstrated that a CBT‐based intervention targeting depression improved ART adherence and increased odds of having an undetectable viral load relative to control [116]. Specific treatment components of these interventions include CBT strategies like problem‐solving [117], behavioural activation [116], approach‐oriented coping skills training [118] and cognitive restructuring [116]. These components can then be packaged and assessed for feasibility, acceptability and signals of preliminary efficacy, both for mental health symptom reduction and increased PrEP adherence/persistence.

The designs of HIV care engagement interventions that target mental health could also be utilized. Task‐sharing to lay counsellors, or peer mothers, may help manage the challenges posed by the limited availability of specialized mental health personnel [119]. Training lay counsellors to address the mental health barriers to PrEP use will build capacity and increase the sustainability of these programmes in ANC, providing critical support for women as they transition to postpartum care, where the focus shifts to the infant. It may be particularly meaningful for PPW to engage with other women who have successfully navigated mental health barriers to PrEP use; this approach was successful in a peer‐run substance use reduction programme that increased engagement in HIV care. Stepped care designs may also prove viable. With the limited availability of psychopharmacology, stepped‐care approaches may begin with brief counselling that targets underlying vulnerabilities across disorders and then escalates to additional treatment and/or a prescription only if symptom reductions and increases in PrEP adherence are not observed. Trauma‐informed HIV care interventions [120] could be adapted to support adherence to PrEP, and dyadic designs could engage both partners while emphasizing the joint benefits of continued PrEP use (e.g. increased intimacy and improved communication) [121]. Finally, integrating brief mental health counselling with PrEP counselling—as has been proposed for HIV primary care [122, 123, 124]—may be feasible in ANC, especially for women who initiate PrEP during pregnancy.

## 3 CONCLUSIONS

We advocate that a comprehensive programme to optimize PrEP implementation in PPW should: (1) include mental health screening tools; (2) identify underlying mechanisms of mental health challenges that are associated with PrEP non‐adherence; and (3) test efficient interventions that target those mechanisms, leveraging existing treatments, empirically‐based strategies and delivery approaches. Although we focus primarily on oral, daily PrEP, mental health challenges will likely also compromise the consistent use of other PrEP agents; as such, we encourage all PrEP implementation research to assess and address mental health, regardless of PrEP formulation. Given the constellation of intersectional and structural challenges that increase the risk for HIV among PPW, interventions to optimize the PrEP cascade during pregnancy and postpartum may not be fully effective or sustainable until they address mental health.

## COMPETING INTERESTS

The authors declare that they have no competing interests.

## AUTHORS’ CONTRIBUTIONS

AMS developed the idea and initiated the collaboration for this manuscript. AMS drafted the paper with critical feedback and guidance from CO, LK, DLJD, LM, JAJ, KHM, LGB and CP. All authors read and approved this article.

## FUNDING

AMS's time was supported by a National Institute on Mental Health Award, K23MH131438 (PI A. Stanton).

## Data Availability

Data sharing is not applicable to this article as no new data were created or analyzed in this study.

## References

[1] UNAIDS . Miles to go: closing gaps, breaking barriers, righting injustices. Global AIDS Update 2018. Geneva: UNAIDS; 2018.

[2] Thomson KA , Hughes J , Baeten JM , John‐Stewart G , Celum C , Cohen CR , et al. Increased risk of HIV acquisition among women throughout pregnancy and during the postpartum period: a prospective Per‐Coital‐Act analysis among women with HIV‐infected partners. J Infect Dis. 2018;218(1):16–25. 29514254 10.1093/infdis/jiy113 PMC5989601

[3] Luppi P. How immune mechanisms are affected by pregnancy. Vaccine. 2003;21(24):3352–7. 12850338 10.1016/s0264-410x(03)00331-1

[4] Hapgood JP , Kaushic C , Hel Z. Hormonal contraception and HIV‐1 acquisition: biological mechanisms. Endocr Rev. 2018;39(1):36–78. 29309550 10.1210/er.2017-00103 PMC5807094

[5] Groer ME , Jevitt C , Ji M . Immune changes and dysphoric moods across the postpartum. Am J Reprod Immunol. 2015;73(3):193–8. 25227158 10.1111/aji.12322 PMC4323631

[6] Graybill LA , Kasaro M , Freeborn K , Walker JS , Poole C , Powers KA , et al. Incident HIV among pregnant and breast‐feeding women in sub‐Saharan Africa: a systematic review and meta‐analysis. AIDS. 2020;34(5):761–76. 32167990 10.1097/QAD.0000000000002487 PMC7275092

[7] Mofenson LM. Risk of HIV acquisition during pregnancy and postpartum: a call for action. J Infect Dis. 2018;218(1):1–4. 29506075 10.1093/infdis/jiy118

[8] Keating MA , Hamela G , Miller WC , Moses A , Hoffman IF , Hosseinipour MC. High HIV incidence and sexual behavior change among pregnant women in Lilongwe, Malawi: implications for the risk of HIV acquisition. PLoS One. 2012;7(6):e39109. 22768063 10.1371/journal.pone.0039109 PMC3387180

[9] Gray RH , Li X , Kigozi G , Serwadda D , Brahmbhatt H , Wabwire‐Mangen F , et al. Increased risk of incident HIV during pregnancy in Rakai, Uganda: a prospective study. Lancet. 2005;366(9492):1182–8. 16198767 10.1016/S0140-6736(05)67481-8

[10] Teasdale CA , Abrams EJ , Chiasson MA , Justman J , Blanchard K , Jones HE. Sexual risk and intravaginal practice behavior changes during pregnancy. Arch Sex Behav. 2017;46(2):539–48. 27600836 10.1007/s10508-016-0818-z

[11] Joseph Davey D , Farley E , Gomba Y , Coates T , Myer L . Sexual risk during pregnancy and postpartum periods among HIV‐infected and –uninfected South African women: implications for primary and secondary HIV prevention interventions. PLoS One. 2018;13(3):e0192982. 29509759 10.1371/journal.pone.0192982 PMC5839542

[12] Sheffield JS , Wendel GD , McIntire DD , Norgard MV. The effect of progesterone levels and pregnancy on HIV‐1 coreceptor expression. Reprod Sci. 2009;16(1):20–31. 19144888 10.1177/1933719108325510

[13] Hayer S , DiClemente K , Swartz A , Chihota Z , Colvin CJ , Short SE , et al. Embodiment, agency, unmet need: young women's experiences in the use and non‐use of contraception in Khayelitsha, South Africa. Glob Public Health. 2022;17(6):885–98. 33600727 10.1080/17441692.2021.1882528 PMC8371059

[14] Mofenson LM. Tenofovir pre‐exposure prophylaxis for pregnant and breastfeeding women at risk of HIV infection: the time is now. PLoS Med. 2016;13(9):e1002133. 10.1371/journal.pmed.1002133 27676386 PMC5038968

[15] Mirochnick M , Best BM , Clarke DF. Antiretroviral pharmacology: special issues regarding pregnant women and neonates. Clin Perinatol. 2010;37(4):907–27. 21078458 10.1016/j.clp.2010.08.006

[16] Le Roux SM , Jao J , Brittain K , Phillips TK , Olatunbosun S , Ronan A , et al. Tenofovir exposure in utero and linear growth in HIV exposed, uninfected infants: a prospective study. AIDS. 2017;31(1):97–104. 27898591 10.1097/QAD.0000000000001302 PMC5814299

[17] Jao J , Abrams EJ , Phillips T , Petro G , Zerbe A , Myer L. In utero tenofovir exposure is not associated with fetal long bone growth. Clin Infect Dis. 2016;62(12):1604–9. 27009251 10.1093/cid/ciw159 PMC4885649

[18] Mugwanya KK , Hendrix CW , Mugo NR , Marzinke M , Katabira ET , Ngure K , et al. Pre‐exposure prophylaxis use by breastfeeding HIV‐uninfected women: a prospective short‐term study of antiretroviral excretion in breast milk and infant absorption. PLoS Med. 2016;13(9):e1002132. 27676257 10.1371/journal.pmed.1002132 PMC5038971

[19] Benaboud S , Pruvost A , Coffie PA , Ekouévi DK , Urien S , Arrivé E , et al. Concentrations of tenofovir and emtricitabine in breast milk of HIV‐1‐infected women in Abidjan, Cote d'Ivoire, in the ANRS 12109 TEmAA Study, Step 2. Antimicrob Agents Chemother. 2011;55(3):1315–7. 21173182 10.1128/AAC.00514-10 PMC3067089

[20] Joseph Davey DL , Pintye J , Baeten JM , Aldrovandi G , Baggaley R , Bekker LG , et al. PrEP in Pregnancy Working Group. Emerging evidence from a systematic review of safety of pre‐exposure prophylaxis for pregnant and postpartum women: where are we now and where are we heading? J Int AIDS Soc. 2020;23(1):e25426. 31912985 10.1002/jia2.25426 PMC6948023

[21] World Health Organization . WHO technical brief: preventing HIV during pregnancy and breastfeeding in the context of PrEP. World Health Organization; 2017.

[22] Grant H , Gomez GB , Kripke K , Barnabas RV , Watts C , Medley GF , et al. Time to scale up preexposure prophylaxis beyond the highest‐risk populations? Modeling insights from high‐risk women in sub‐Saharan Africa. Sex Transm Dis. 2020;47(11):767–77. 33044426 10.1097/OLQ.0000000000001253

[23] Davey DJ , Bekker LG , Gomba Y , Coates T , Myer L , Johnson LF. Modelling the potential impact of providing pre‐exposure prophylaxis (PrEP) in pregnant and breastfeeding women in South Africa. AIDS. 2019;33(8):1391. 30950882 10.1097/QAD.0000000000002221 PMC6561341

[24] Kramer MS , Kakuma R. Optimal duration of exclusive breastfeeding. Cochrane Database Syst Rev. 2012;2012(8):CD003517. 10.1002/14651858.CD003517.pub2 PMC7154583 22895934

[25] Bhutta ZA , Ahmed T , Black RE , Cousens S , Dewey K , Giugliani E , et al. What works? Interventions for maternal and child undernutrition and survival. Lancet. 2008;371(9610):417–40. 18206226 10.1016/S0140-6736(07)61693-6

[26] Smith HA , Becker GE. Early additional food and fluids for healthy breastfed full‐term infants. Cochrane Database Syst Rev. 2016, Issue 8. Art. No.: CD006462. 27574798 10.1002/14651858.CD006462.pub4 PMC8588276

[27] World Health Organization . Global strategy for infant and young child feeding. Geneva: World Health Organization; 2003.

[28] World Health Organization . Breastfeeding: recommendations. [cited 2020 Sep 2]. Available from: https://www.who.int/westernpacific/health‐topics/breastfeeding

[29] le Roux SM , Abrams EJ , Nguyen KK , Myer L. HIV incidence during breastfeeding and mother‐to‐child transmission in Cape Town, South Africa. AIDS. 2019;33(8):1399–401. 30932960 10.1097/QAD.0000000000002224

[30] Johnson L , Dorrington R. Thembisa version 4.1: a model for evaluating the impact of HIV/AIDS in South Africa. [cited 2019 July 22] Available at: https://thembisa.org/content/downloadPage/Thembisa4_1report.

[31] Johnson LF , Patrick M , Stephen C , Patten G , Dorrington RE , Maskew M , et al. Steep declines in pediatric AIDS mortality in South Africa, despite poor progress toward pediatric diagnosis and treatment targets. Pediatr Infect Dis J. 2020;39(9):843–8. 32433224 10.1097/INF.0000000000002680 PMC7958302

[32] Joseph Davey DL , Mvududu R , Mashele N , Lesosky M , Khadka N , Bekker L , et al. Early pre‐exposure prophylaxis (PrEP) initiation and continuation among pregnant and postpartum women in antenatal care in Cape Town, South Africa. J Int AIDS Soc. 2022;25(2):e25866. 35138678 10.1002/jia2.25866 PMC8826542

[33] Matthews LT , Jaggernath M , Kriel Y , Smith PM , O'Neil K , Haberer JE , et al. Protocol for a longitudinal study to evaluate the use of tenofovir‐based PrEP for safer conception and pregnancy among women in South Africa. BMJ Open. 2019;9(7):e027227. 10.1136/bmjopen-2018-027227 PMC6661571 31350241

[34] Kinuthia J , Pintye J , Abuna F , Mugwanya KK , Lagat H , Onyango D , et al. Pre‐exposure prophylaxis uptake and early continuation among pregnant and post‐partum women within maternal and child health clinics in Kenya: results from an implementation programme. Lancet HIV. 2020;7(1):e38–48. 31813837 10.1016/S2352-3018(19)30335-2 PMC11498332

[35] Tessema ZT , Teshale AB , Tesema GA , Tamirat KS. Determinants of completing recommended antenatal care utilization in sub‐Saharan from 2006 to 2018: evidence from 36 countries using Demographic and Health Surveys. BMC Pregnancy Childbirth. 2021;21(1):1–12. 33676440 10.1186/s12884-021-03669-w PMC7937261

[36] Celum C , Hosek S , Tsholwana M , Kassim S , Mukaka S , Dye BJ , et al. PrEP uptake, persistence, adherence, and effect of retrospective drug level feedback on PrEP adherence among young women in southern Africa: results from HPTN 082, a randomized controlled trial. PLoS Med. 2021;18(6):e1003670. 34143779 10.1371/journal.pmed.1003670 PMC8253429

[37] Cottrell ML , Yang KH , Prince HMA , Sykes C , White N , Malone S , et al. Predicting effective Truvada® PrEP dosing strategies with a novel PK‐PD model incorporating tissue active metabolites and endogenous nucleotides (EN). AIDS Res Hum Retroviruses. 2014;30(S1):A60.

[38] Patterson KB , Prince HA , Kraft E , Jenkins AJ , Shaheen NJ , Rooney JF , et al. Penetration of tenofovir and emtricitabine in mucosal tissues: implications for prevention of HIV‐1 transmission. Sci Transl Med. 2011;3(112):112re4. 10.1126/scitranslmed.3003174 PMC3483088 22158861

[39] Anderson PL , Stranix‐Chibanda L , Huang S , Hosek S , Kacanek D , Nematadzira T , et al. TFV‐DP in DBS for pregnant/postpartum adolescent and young women on PrEP in Africa. 2020.

[40] Stranix‐Chibanda L , Anderson PL , Kacanek D , Hosek S , Huang S , Nematadzira TG , et al. Tenofovir diphosphate concentrations in dried blood spots from pregnant and postpartum adolescent and young women receiving daily observed pre‐exposure prophylaxis in sub‐Saharan Africa. Clin Infect Dis. 2021;73(7):e1893–900. 33341883 10.1093/cid/ciaa1872 PMC8492211

[41] Maria P , Anderson PL , Hendrix CW , Heffron R , Mugwanya K , Haberer JE , et al. Tenofovir and tenofovir‐diphosphate concentrations during pregnancy among HIV‐uninfected women using oral pre‐exposure prophylaxis. AIDS. 2018;32(13):1891. 29894385 10.1097/QAD.0000000000001922 PMC6061961

[42] Haberer JE , Bangsberg DR , Baeten JM , Curran K , Koechlin F , Amico KR , et al. Defining success with HIV pre‐exposure prophylaxis: a prevention‐effective adherence paradigm. AIDS. 2015;29(11):1277–85. 26103095 10.1097/QAD.0000000000000647 PMC4480436

[43] Haberer JE. Current concepts for PrEP adherence: in the PrEP revolution; from clinical trials to routine practice. Curr Opin HIV AIDS. 2016;11(1):10. 26633638 10.1097/COH.0000000000000220 PMC4801217

[44] Mugwanya KK , Pintye J , Kinuthia J , Abuna F , Lagat H , Begnel ER , et al. Integrating preexposure prophylaxis delivery in routine family planning clinics: a feasibility programmatic evaluation in Kenya. PLoS Med. 2019;16(9):e1002885. 31479452 10.1371/journal.pmed.1002885 PMC6719826

[45] Joseph Davey D , Mvududu R , Mashele N , Lesosky M , Bekker LG , Gorbach P , et al. High initiation and persistence on pre‐exposure prophylaxis (PrEP) in HIV‐uninfected pregnant women in Cape Town, South Africa. 2021. Accessed on February 1, 2022. Available from: https://programme.hivr4p.org/Abstract/Abstract/1416

[46] Pintye J , O'Malley G , Kinuthia J , Abuna F , Escudero JN , Mugambi M , et al. Influences on early discontinuation and persistence of daily oral PrEP use among Kenyan adolescent girls and young women: a qualitative evaluation from a PrEP implementation program. J Acquir Immune Defic Syndr. 2021;86(4):e83–9. 33273211 10.1097/QAI.0000000000002587 PMC8935942

[47] Roberts ST , Haberer J , Celum C , Mugo N , Ware NC , Cohen CR , et al. Intimate partner violence and adherence to HIV pre‐exposure prophylaxis (PrEP) in African women in HIV serodiscordant relationships: a prospective cohort study. J Acquir Immune Defic Syndr. 2016;73(3):313–22. 27243900 10.1097/QAI.0000000000001093 PMC5065369

[48] Velloza J , Baeten JM , Haberer J , Ngure K , Irungu E , Mugo NR , et al. Effect of depression on adherence to oral PrEP among men and women in East Africa. J Acquir Immune Defic Syndr. 2018;79(3):330–8. 30063651 10.1097/QAI.0000000000001821 PMC6295199

[49] Velloza J , Heffron R , Amico KR , Rowhani‐Rahbar A , Hughes JP , Li M , et al. The effect of depression on adherence to HIV pre‐exposure prophylaxis among high‐risk South African women in HPTN 067/ADAPT. AIDS Behav. 2020;24(7):2178–87. 31955360 10.1007/s10461-020-02783-8 PMC7319871

[50] Palanee‐Phillips T , Roberts ST , Reddy K , Govender V , Naidoo L , Siva S , et al. Impact of partner‐related social harms on women's adherence to the dapivirine vaginal ring during a phase III trial. J Acquir Immune Defic Syndr. 2018;79(5):580–9. 30239426 10.1097/QAI.0000000000001866 PMC6231955

[51] Cabral A , Baeten JM , Ngure K , Velloza J , Odoyo J , Haberer JE , et al. Intimate partner violence and self‐reported pre‐exposure prophylaxis interruptions among HIV‐negative partners in HIV serodiscordant couples in Kenya and Uganda. J Acquir Immune Defic Syndr. 2018;77(2):154–9. 29076883 10.1097/QAI.0000000000001574 PMC5762272

[52] Celum CL , Delany‐Moretlwe S , McConnell M , van Rooyen H , Bekker LG , Kurth A , et al. Rethinking HIV prevention to prepare for oral PrEP implementation for young African women. J Int AIDS Soc. 2015;18(4Suppl 3):20227. 26198350 10.7448/IAS.18.4.20227 PMC4509892

[53] Celum CL , Delany‐Moretlwe S , Baeten JM , van der Straten A , Hosek S , Bukusi EA , et al. HIV pre‐exposure prophylaxis for adolescent girls and young women in Africa: from efficacy trials to delivery. J Int AIDS Soc. 2019;22(Suppl 4):e25298. 31328444 10.1002/jia2.25298 PMC6643076

[54] van der Straten A , Stadler J , Montgomery E , Hartmann M , Magazi B , Mathebula F , et al. Women's experiences with oral and vaginal pre‐exposure prophylaxis: the VOICE‐C qualitative study in Johannesburg, South Africa. PLoS One. 2014;9(2):e89118. 24586534 10.1371/journal.pone.0089118 PMC3931679

[55] Hartmann M , McConnell M , Bekker LG , Celum C , Bennie T , Zuma J , et al. Motivated reasoning and HIV risk? Views on relationships, trust, and risk from young women in Cape Town, South Africa, and implications for oral PrEP. AIDS Behav. 2018;22(11):3468–79. 29404757 10.1007/s10461-018-2044-2 PMC6077112

[56] Amico KR , Wallace M , Bekker LG , Roux S , Atujuna M , Sebastian E , et al. Experiences with HPTN 067/ADAPT study‐provided open‐label PrEP among women in Cape Town: facilitators and barriers within a mutuality framework. AIDS Behav. 2017;21(5):1361–75. 27317411 10.1007/s10461-016-1458-y PMC5378745

[57] Beesham I , Dovel K , Mashele N , Bekker LG , Gorbach P , Coates TJ , et al. Barriers to oral HIV pre‐exposure prophylaxis (PrEP) adherence among pregnant and post‐partum women from Cape Town, South Africa. AIDS Behav. 2022;22:1–9. 10.1007/s10461-022-03652-2 PMC8938734 35316471

[58] Atukunda EC , Owembabazi M , Pratt MC , Psaros C , Muyindike W , Chitneni P , et al. A qualitative exploration to understand barriers and facilitators to daily oral PrEP uptake and sustained adherence among HIV‐negative women planning for or with pregnancy in rural Southwestern Uganda. J Int AIDS Soc. 2022;25(3):e25894. 35324081 10.1002/jia2.25894 PMC8944216

[59] Patel P , Thiagarajah S , Ford S , Margolis DA , Romach B , Baker M , et al. Cabotegravir pharmacokinetic tail in pregnancy and neonatal outcomes. 2020.

[60] Makanani B , Balkus JE , Jiao Y , Noguchi LM , Palanee‐Phillips T , Mbilizi Y , et al. Pregnancy and infant outcomes among women using the dapivirine vaginal ring in early pregnancy. J Acquir Immune Defic Syndr. 2018;79(5):566. 30383589 10.1097/QAI.0000000000001861 PMC6231990

[61] Joseph Davey DL , Bekker LG , Bukusi EA , Chi BH , Delany‐Moretlwe S , Goga A , et al. Where are the pregnant and breastfeeding women in new pre‐exposure prophylaxis trials? The imperative to overcome the evidence gap. Lancet HIV. 2022;9(3):e214–22. 35090604 10.1016/S2352-3018(21)00280-0 PMC9178651

[62] Shamu S , Abrahams N , Temmerman M , Musekiwa A , Zarowsky C. A systematic review of African studies on intimate partner violence against pregnant women: prevalence and risk factors. PLoS One. 2011;6(3):e17591. 21408120 10.1371/journal.pone.0017591 PMC3050907

[63] Tsai AC , Tomlinson M , Comulada WS , Rotheram‐Borus MJ. Intimate partner violence and depression symptom severity among South African women during pregnancy and postpartum: population‐based prospective cohort study. PLoS Med. 2016;13(1):e1001943. 26784110 10.1371/journal.pmed.1001943 PMC4718639

[64] Kaminer D , Grimsrud A , Myer L , Stein DJ , Williams DR. Risk for post‐traumatic stress disorder associated with different forms of interpersonal violence in South Africa. Soc Sci Med. 2008;67(10):1589–95. 18774211 10.1016/j.socscimed.2008.07.023 PMC2610682

[65] Velloza J , Hosek S , Donnell D , Anderson PL , Chirenje M , Mgodi N , et al. Assessing longitudinal patterns of depressive symptoms and the influence of symptom trajectories on HIV pre‐exposure prophylaxis adherence among adolescent girls in the HPTN 082 randomized controlled trial. J Int AIDS Soc. 2021;24:e25731. 34164929 10.1002/jia2.25731 PMC8222844

[66] Sawyer A , Ayers S , Smith H. Pre‐ and postnatal psychological wellbeing in Africa: a systematic review. J Affect Disord. 2010;123(1–3):17–29. 19635636 10.1016/j.jad.2009.06.027

[67] Roberts KJ , Smith C , Cluver L , Toska E , Sherr L. Understanding mental health in the context of adolescent pregnancy and HIV in sub‐Saharan Africa: a systematic review identifying a critical evidence gap. AIDS Behav. 2021;25(7):2094–107. 33452658 10.1007/s10461-020-03138-z PMC7810185

[68] Shamu S , Zarowsky C , Roelens K , Temmerman M , Abrahams N. High‐frequency intimate partner violence during pregnancy, postnatal depression and suicidal tendencies in Harare, Zimbabwe. Gen Hosp Psychiatry. 2016;38:109–14. 26607330 10.1016/j.genhosppsych.2015.10.005

[69] Iyun V , Brittain K , Phillips TK , Le Roux S , McIntyre JA , Zerbe A , et al. Prevalence and determinants of unplanned pregnancy in HIV‐positive and HIV‐negative pregnant women in Cape Town, South Africa: a cross‐sectional study. BMJ Open. 2018;8(4):e019979. 10.1136/bmjopen-2017-019979 PMC5892733 29615449

[70] Rochat TJ , Bland RM , Tomlinson M , Stein A. Suicide ideation, depression and HIV among pregnant women in rural South Africa. 2013.

[71] Choi KW , Smit JA , Coleman JN , Mosery N , Bangsberg DR , Safren SA , et al. Mapping a syndemic of psychosocial risks during pregnancy using network analysis. Int J Behav Med. 2019;26(2):207–16. 30805768 10.1007/s12529-019-09774-7 PMC6628702

[72] Duby Z , Appollis TM , Jonas K , Maruping K , Dietrich J , LoVette A , et al. “As a young pregnant girl… the challenges you face”: exploring the intersection between mental health and sexual and reproductive health amongst adolescent girls and young women in South Africa. AIDS Behav. 2021;25(2):344–53. 32683636 10.1007/s10461-020-02974-3 PMC7368608

[73] Singer M. Introduction to syndemics: a critical systems approach to public and community health. John Wiley & Sons; 2009.

[74] Mendenhall E , Singer M. What constitutes a syndemic? Methods, contexts, and framing from 2019. Curr Opin HIV AIDS. 2020;15(4):213–7. 32412998 10.1097/COH.0000000000000628

[75] Stringer EM , Meltzer‐Brody S , Kasaro M , Stuebe AM , Wiegand S , Paul R , et al. Depression, pregnancy, and HIV: the case to strengthen mental health services for pregnant and post‐partum women in sub‐Saharan Africa. Lancet Psychiatry. 2014;1(2):159–62. 26360580 10.1016/S2215-0366(14)70273-1

[76] Ng'oma M , Bitew T , Kaiyo‐Utete M , Hanlon C , Honikman S , Stewart RC. Perinatal mental health around the world: priorities for research and service development in Africa. BJPsych Int. 2020;17(3):56–9. 34287427 10.1192/bji.2020.16 PMC8281281

[77] Pintye J , Davey DLJ , Wagner AD , John‐Stewart G , Baggaley R , Bekker LG , et al. Defining gaps in pre‐exposure prophylaxis delivery for pregnant and post‐partum women in high‐burden settings using an implementation science framework. Lancet HIV. 2020;7(8):e582–92. 32763221 10.1016/S2352-3018(20)30102-8 PMC7587402

[78] Howard LM. What does excellence in perinatal mental health look like. 2016.

[79] Siu AL , Bibbins‐Domingo K , Grossman DC , Baumann LC , Davidson KW , Ebell M , et al. Screening for depression in adults: US Preventive Services Task Force recommendation statement. JAMA. 2016;315(4):380–7. 26813211 10.1001/jama.2015.18392

[80] Thombs BD , Coyne JC , Cuijpers P , De Jonge P , Gilbody S , Ioannidis JP , et al. Rethinking recommendations for screening for depression in primary care. CMAJ. 2012;184(4):413–8. 21930744 10.1503/cmaj.111035 PMC3291670

[81] Thombs BD , Arthurs E , Coronado‐Montoya S , Roseman M , Delisle VC , Leavens A , et al. Depression screening and patient outcomes in pregnancy or postpartum: a systematic review. J Psychosom Res. 2014;76(6):433–46. 24840137 10.1016/j.jpsychores.2014.01.006

[82] Brown S , Sprague C. Health care providers’ perceptions of barriers to perinatal mental healthcare in South Africa. BMC Public Health. 2021;21(1):1–13. 34670531 10.1186/s12889-021-11954-8 PMC8528557

[83] Lawrie T , Hofmeyr G , De Jager M , Berk M. Validation of the Edinburgh Postnatal Depression Scale on a cohort of South African women. S Afr Med J. 1998;88(10):1340–4. 9807193

[84] Kroenke K , Spitzer RL , Williams JB. The PHQ‐9: validity of a brief depression severity measure. J Gen Intern Med. 2001;16(9):606–13. 11556941 10.1046/j.1525-1497.2001.016009606.x PMC1495268

[85] Chibanda D , Mangezi W , Tshimanga M , Woelk G , Rusakaniko P , Stranix‐Chibanda L , et al. Validation of the Edinburgh Postnatal Depression Scale among women in a high HIV prevalence area in urban Zimbabwe. Arch Womens Ment Health. 2010;13(3):201–6. 19760051 10.1007/s00737-009-0073-6

[86] Stewart RC , Umar E , Tomenson B , Creed F . Validation of screening tools for antenatal depression in Malawi—a comparison of the Edinburgh Postnatal Depression Scale and Self Reporting Questionnaire. J Affect Disord. 2013;150(3):1041–7. 23769290 10.1016/j.jad.2013.05.036

[87] Abrahams Z , Schneider M , Field S , Honikman S. Validation of a brief mental health screening tool for pregnant women in a low socio‐economic setting. BMC Psychol. 2019;7(1):1–11. 31818326 10.1186/s40359-019-0355-3 PMC6902551

[88] Sweetland AC , Belkin GS , Verdeli H. Measuring depression and anxiety in sub‐Saharan Africa. Depress Anxiety. 2014;31(3):223–32. 23780834 10.1002/da.22142 PMC4109689

[89] Larsen A , Pintye J , Bhat A , Kumar M , Kinuthia J , Collins PY , et al. Is there an optimal screening tool for identifying perinatal depression within clinical settings of sub‐Saharan Africa? SSM‐Ment Health. 2021;1:100015. 10.1016/j.ssmmh.2021.100015 PMC13288248 42344140

[90] Van Heyningen T , Honikman S , Tomlinson M , Field S , Myer L. Comparison of mental health screening tools for detecting antenatal depression and anxiety disorders in South African women. PLoS One. 2018;13(4):e0193697. 29668725 10.1371/journal.pone.0193697 PMC5906008

[91] Marsay C , Manderson L , Subramaney U. Validation of the Whooley questions for antenatal depression and anxiety among low‐income women in urban South Africa. South Afr J Psychiatry. 2017;23(1):1–7. 10.4102/sajpsychiatry.v23i0.1013 PMC6138202 30263185

[92] Kagee A , Tsai AC , Lund C , Tomlinson M. Screening for common mental disorders in low resource settings: reasons for caution and a way forward. Int Health. 2013;5(1):11–4. 23580905 10.1093/inthealth/ihs004 PMC3619733

[93] Patel V , Simunyu E , Gwanzura F , Lewis G , Mann A. The Shona Symptom Questionnaire: the development of an indigenous measure of common mental disorders in Harare. Acta Psychiatr Scand. 1997;95(6):469–75. 9242841 10.1111/j.1600-0447.1997.tb10134.x

[94] Fernando S , Brown T , Datta K , Chidhanguro D , Tavengwa NV , Chandna J , et al. The Friendship Bench as a brief psychological intervention with peer support in rural Zimbabwean women: a mixed methods pilot evaluation. Glob Ment Health. 2021;8:e31. 10.1017/gmh.2021.32 PMC8392686 34513000

[95] Verhey R , Chibanda D , Gibson L , Brakarsh J , Seedat S. Validation of the posttraumatic stress disorder checklist ‐ 5 (PCL‐5) in a primary care population with high HIV prevalence in Zimbabwe. BMC Psychiatry. 2018;18(1):109. 29685117 10.1186/s12888-018-1688-9 PMC5913864

[96] Andersen LS , Joska JA , Magidson JF , O'Cleirigh C , Lee JS , Kagee A , et al. Detecting depression in people living with HIV in South Africa: the factor structure and convergent validity of the South African Depression Scale (SADS). AIDS Behav. 2020;24(8):2282–9. 31965430 10.1007/s10461-020-02787-4 PMC8021389

[97] Akena D , Joska J , Stein DJ. Sensitivity and specificity of the Akena Visual Depression Inventory (AViDI‐18) in Kampala (Uganda) and Cape Town (South Africa). Br J Psychiatry J Ment Sci. 2018;212(5):301–7. 10.1192/bjp.2018.9 29554984

[98] Hayes SC , Wilson KG , Gifford EV , Follette VM , Strosahl K. Experimental avoidance and behavioral disorders: a functional dimensional approach to diagnosis and treatment. J Consult Clin Psychol. 1996;64(6):1152–68. 8991302 10.1037//0022-006x.64.6.1152

[99] Buyck D. Depression in context: strategies for guided action. Prim Care Companion J Clin Psychiatry. 2002;4(5):201.

[100] O'Cleirigh C , Safren SA , Mayer KH. The pervasive effects of childhood sexual abuse: challenges for improving HIV prevention and treatment interventions. J Acquir Immune Defic Syndr. 2012;59(4):331–4. 22293545 10.1097/QAI.0b013e31824aed80 PMC3657844

[101] Leyro TM , Zvolensky MJ , Bernstein A. Distress tolerance and psychopathological symptoms and disorders: a review of the empirical literature among adults. Psychol Bull. 2010;136(4):576–600. 20565169 10.1037/a0019712 PMC2891552

[102] Tull MT , Gratz KL , Salters K , Roemer L. The role of experiential avoidance in posttraumatic stress symptoms and symptoms of depression, anxiety, and somatization. J Nerv Ment Dis. 2004;192(11):754–61. 15505519 10.1097/01.nmd.0000144694.30121.89

[103] Trew JL. Exploring the roles of approach and avoidance in depression: an integrative model. Clin Psychol Rev. 2011;31(7):1156–68. 21855826 10.1016/j.cpr.2011.07.007

[104] Akbari M , Hosseini ZS , Seydavi M , Zegel M , Zvolensky MJ , Vujanovic AA. Distress tolerance and posttraumatic stress disorder: a systematic review and meta‐analysis. Cogn Behav Ther. 2022;51(1):42–71. 34279189 10.1080/16506073.2021.1942541

[105] Lass ANS , Winer ES. Distress tolerance and symptoms of depression: a review and integration of literatures. Clin Psychol Sci Pract. 2020;27(3):e12336. 10.1111/cpsp.12336

[106] Boden MT , Kulkarni M , Shurick A , Bonn‐Miller MO , Gross JJ. Responding to trauma and loss: an emotion regulation perspective. Martha Kent, Mary C. Davis, and John W. Reich. (ed.). In: The resilience handbook: approaches to stress and trauma. New York: Routledge/Taylor & Francis Group; 2014. p. 86–99.

[107] Joormann J , Stanton CH. Examining emotion regulation in depression: a review and future directions. Behav Res Ther. 2016;86:35–49. 27492851 10.1016/j.brat.2016.07.007

[108] Allen LB , McHugh RK , Barlow DH. Emotional disorders: a unified protocol. 2008.

[109] Murray LK , Dorsey S , Haroz E , Lee C , Alsiary MM , Haydary A , et al. A common elements treatment approach for adult mental health problems in low‐and middle‐income countries. Cogn Behav Pract. 2014;21(2):111–23. 25620867 10.1016/j.cbpra.2013.06.005 PMC4304666

[110] Chorpita BF , Daleiden EL. Mapping evidence‐based treatments for children and adolescents: application of the distillation and matching model to 615 treatments from 322 randomized trials. J Consult Clin Psychol. 2009;77(3):566. 19485596 10.1037/a0014565

[111] Mansell W , Harvey A , Watkins ER , Shafran R. Cognitive behavioral processes across psychological disorders: a review of the utility and validity of the transdiagnostic approach. Int J Cogn Ther. 2008;1(3):181–91.

[112] McHugh RK , Murray HW , Barlow DH. Balancing fidelity and adaptation in the dissemination of empirically‐supported treatments: the promise of transdiagnostic interventions. Behav Res Ther. 2009;47(11):946–53. 19643395 10.1016/j.brat.2009.07.005 PMC2784019

[113] Weisz JR , Krumholz LS , Santucci L , Thomassin K , Ng MY. Shrinking the gap between research and practice: tailoring and testing youth psychotherapies in clinical care contexts. Annu Rev Clin Psychol. 2015;11:139–63. 25822345 10.1146/annurev-clinpsy-032814-112820

[114] Sikkema KJ , Mulawa MI , Robertson C , Watt MH , Ciya N , Stein DJ , et al. Improving AIDS Care After Trauma (ImpACT): pilot outcomes of a coping intervention among HIV‐infected women with sexual trauma in South Africa. AIDS Behav. 2018;22(3):1039–52. 29270789 10.1007/s10461-017-2013-1 PMC5828984

[115] Psaros C , Stanton AM , Raggio GA , Mosery N , Goodman GR , Briggs ES , et al. Optimizing PMTCT adherence by treating depression in perinatal women with HIV in South Africa: a pilot randomized controlled trial. Int J Behav Med. 2022;8:1–5. 10.1007/s12529-022-10071-z PMC9452601 35260947

[116] Safren SA , O'Cleirigh C , Andersen LS , Magidson JF , Lee JS , Bainter SA , et al. Treating depression and improving adherence in HIV care with task‐shared cognitive behavioural therapy in Khayelitsha, South Africa: a randomized controlled trial. J Int AIDS Soc. 2021;24(10):e25823. 34708929 10.1002/jia2.25823 PMC8552453

[117] Abas M , Bowers T , Manda E , Cooper S , Machando D , Verhey R , et al. ‘Opening up the mind’: problem‐solving therapy delivered by female lay health workers to improve access to evidence‐based care for depression and other common mental disorders through the Friendship Bench Project in Zimbabwe. Int J Ment Health Syst. 2016;10(1):39. 27175215 10.1186/s13033-016-0071-9 PMC4865009

[118] Sikkema KJ , Choi KW , Robertson C , Knettel BA , Ciya N , Knippler ET , et al. Development of a coping intervention to improve traumatic stress and HIV care engagement among South African women with sexual trauma histories. Eval Program Plann. 2018;68:148–56. 29597104 10.1016/j.evalprogplan.2018.02.007 PMC5953816

[119] Kakuma R , Minas H , van Ginneken N , Dal Poz MR , Desiraju K , Morris JE , et al. Human resources for mental health care: current situation and strategies for action. Lancet. 2011;378(9803):1654–63. 22008420 10.1016/S0140-6736(11)61093-3

[120] Brown MJ , Adeagbo O. Trauma‐informed HIV care interventions: towards a holistic approach. Curr HIV/AIDS Rep. 2022;19(3):177–83. 35353271 10.1007/s11904-022-00603-3 PMC10084732

[121] Stanton AM , Bwana M , Owembabazi M , Atukunda E , Musinguzi E , Ezegbe H , et al. Sexual and relationship benefits of a safer conception intervention among men with HIV who seek to have children with serodifferent partners in Uganda. AIDS Behav. 2022;26(6):1841–52. 34796420 10.1007/s10461-021-03533-0 PMC9050835

[122] Remien RH , Stirratt MJ , Nguyen N , Robbins RN , Pala AN , Mellins CA. Mental health and HIV/AIDS: the need for an integrated response. AIDS. 2019;33(9):1411–20. 30950883 10.1097/QAD.0000000000002227 PMC6635049

[123] Myers B , Joska J , Lund C , Levitt N , Butler C , Naledi T , et al. Patient preferences for the integration of mental health counseling and chronic disease care in South Africa. Patient Prefer Adherence. 2018;12:1797–803. 30271123 10.2147/PPA.S176356 PMC6154740

[124] Udedi M , Stockton MA , Kulisewa K , Hosseinipour MC , Gaynes BN , Mphonda SM , et al. Integrating depression management into HIV primary care in central Malawi: the implementation of a pilot capacity building program. BMC Health Serv Res. 2018;18(1):593. 30064418 10.1186/s12913-018-3388-z PMC6069990

